# DATA-DRIVEN APPROACHES TO UNDERSTAND PROTEOMICS SIGNATURES IN DEMENTIA: RESULTS FROM ELSA AND UK BIOBANK

**DOI:** 10.1093/geroni/igae098.2269

**Published:** 2024-12-31

**Authors:** Jessica Gong, Dylan Williams, Andrew Steptoe

**Affiliations:** University College London, London, England, United Kingdom; University College London, London, England, United Kingdom; University College London, London, England, United Kingdom

## Abstract

Novel blood-based protein markers are crucial for understanding dementia’s biological mechanisms. Undertaking a data-driven, proteome-wide approach to understand the protein signatures in dementia, this study used the English Longitudinal Study of Ageing (ELSA) as the discovery cohort, and subsequently the UK Biobank (UKB) as the validation cohort, to estimate the associations between proteins and the risk of incident all-cause dementia (ACD), and dementia subtypes. Bidirectional Mendelian randomization (MR) and drug target MR were employed to establish causal evidence between proteins and dementia as well as cerebral white matter volume (WMV). Of the 276 proteins in 3249 samples from ELSA (229 incident ACD, 89 Alzheimer’s disease (AD), 41 vascular dementia (VAD)), over a median 9.8-year follow-up, two proteins (NEFL, RPS6KB1) were significant after full adjustments, using Cox Proportional Hazard regression models, with False Discovery Rate p-value set at 0.05 to indicate statistical significance. One protein (MMP12) was found to be associated with VAD. NEFL and RPS6KB1 in combination with demographic predictors (sex, age, education, race), along with APOE 4 genotype and memory score showed good predictive value (Area Under Curve (AUC)=0.87). All associations were replicated in UKB in 52,745 participants (1506 ACD, 732 AD, 281 VAD) over median 13.7-year follow-up. Causal evidence for MR indicated a backward association between dementia and NEFL; drug target MR indicated causal relationships between NEFL and AD and WMV, as well as MMP12 and VAD. This study highlights promising blood-based protein candidates for dementia diagnostics and therapeutic targets, emphasizing the potential for improving patient care.

